# Conceptual models for Mental Distress among HIV-infected and uninfected individuals: A contribution to clinical practice and research in primary-health-care centers in Zambia

**DOI:** 10.1186/1472-6963-11-7

**Published:** 2011-01-10

**Authors:** Peter J Chipimo, Mary Tuba, Knut Fylkesnes

**Affiliations:** 1 University of Zambia, School of Medicine, Department of Community Medicine, Box 50110, Lusaka, Zambia; 2 University of Bergen, Centre for International Health, Faculty of Medicine and Dentistry, 5020, Bergen, Norway

## Abstract

**Background:**

Mental distress is common in primary care and overrepresented among Human Immunodeficiency virus (HIV)-infected individuals, but access to effective treatment is limited, particularly in developing countries. Explanatory models (EM) are contextualised explanations of illnesses and treatments framed within a given society and are important in understanding an individual's perspective on the illness. Although individual variations are important in determining help-seeking and treatment behaviour patterns, the ability to cope with an illness and quality of life, the role of explanatory models in shaping treatment preferences is undervalued. The aim was to identify explanatory models employed by HIV-infected and uninfected individuals and to compare them with those employed by local health care providers. Furthermore, we aimed to build a theoretical model linking the perception of mental distress to treatment preferences and coping mechanisms.

**Methods:**

Qualitative investigation nested in a cross-sectional validation study of 28 (male and female) attendees at four primary care clinics in Lusaka, Zambia, between December 2008 and May 2009. Consecutive clinic attendees were sampled on random days and conceptual models of mental distress were examined, using semi-structured interviews, in order to develop a taxonomic model in which each category was associated with a unique pattern of symptoms, treatment preferences and coping strategies.

**Results:**

Mental distress was expressed primarily as somatic complaints including headaches, perturbed sleep and autonomic symptoms. Economic difficulties and interpersonal relationship problems were the most common causal models among uninfected individuals. Newly diagnosed HIV patients presented with a high degree of hopelessness and did not value seeking help for their symptoms. Patients not receiving anti-retroviral drugs (ARV) questioned their effectiveness and were equivocal about seeking help. Individuals receiving ARV were best adjusted to their status, expressed hope and valued counseling and support groups. Health care providers reported that 40% of mental distress cases were due to HIV infection.

**Conclusions:**

Patient models concerning mental distress are critical to treatment-seeking decisions and coping mechanisms. Mental health interventions should be further researched and prioritized for HIV-infected individuals.

## Background

Mental disorders contribute substantially to the global burden of disease, accounting for 15% of all disability-adjusted-life-years and up to 30% of those attributable to non-communicable diseases[1,2]. Factors emphasizing the role of poverty including economic deprivation, lack of social support structures, gender disadvantage [3]and medical illnesses are major determinants of mental distress and risk factors for new episodes of affective disorders[3]. There is much debate concerning the most effective way to measure mental distress. Several instruments, most of which are based on self-report systems, have been suggested for detecting mental distress, including the Self-Reporting questionnaire (SRQ), Symptom Check List (SCL) and General Health Questionnaire (GHQ), and the validity of these methods have been published[4-6]. Cultural construction of mental distress is important[7,8]. Debate concerning the significance of cultural construction in relation to mental distress has led to increased interest in explanatory models that focus on mental disorders in different communities[9-11]. Such models influenced the inclusion of an outline for the cultural formulation of "culture-bound syndromes" in the fourth version of the Diagnostic Statistical Manual of Mental Disorders (DSM-IV revised) [12]

In Zambia, literature concerning specific definitions and the perception of mental distress is limited, as mental health is generally not prioritised in terms of service provision [13]. However, rates of mental and emotional illness are thought to be increasing in Zambia owing to socio-economic difficulties that precipitate mental problems including HIV/AIDS, poverty and lack of employment[4,5,14]. Mental illness is generally viewed from two broad perspectives, community and cultural[13]. The community view perceives good mental health or "a sound mind" as the ability to execute roles and responsibilities expected within a given social and cultural context. In contrast, mental illness, whether mild or severe, is associated with disruptive behaviour, straitjackets, and mental institutions[12,13]. In Zambia, cultural beliefs concerning the cause of mental illness centre on possession by spirits or social punishment; many hold the belief that mental illness is caused by witchcraft and therefore cannot be treated by modern medicine but only through traditional means. A lack of proper information and the dominance of misleading presentation have led to a negative portrayal of mental illness, and sufferers are collectively and unjustifiably categorized and rejected, regardless of the form of mental illness [13,15].

Such categorization often leads to mistreatment and isolation of mentally ill individuals [15]. A qualitative study in Zambia revealed that mental health patients utilizing health services felt stigmatized and discriminated against, and a further study investigating the quality of life of women suffering from mental illness revealed similar results[14,15]. The stigma attached to mental illness caused both community and health decision-makers to view sufferers with low regard, leading to stigmatization of families across generations, institutions that provide treatment, psychotropic drugs and mental health workers [15]. Such attitudes are an obstacle to the provision of care as they result in a reluctance to invest resources into mental health care and discrimination in the provision of services for physical illness among those who are mentally ill. The studies outlined above concluded that mental illness is a complex and diverse disorder, and that there is need to employ a multi-dimensional approach for the diagnosis and management of mental illness in public health institutions.

### Explanatory Models

Explanatory models (EM) are defined as an understanding or explanation of episodes of illness and treatment, framed within the context of cultural beliefs and norms of a given society, and employed by all engaged in the clinical process and the interaction between healer and patient that is central to the health care system[14]. Rather than adopting a single explanatory perspective, as is often the case with traditional theories of science, i.e. etiological models, a range of compelling evidence indicates that mental distress involves causal pathways that act both within and outside the individual[12]. Therefore, it is imperative that mental distress is understood in terms of biological, psychological, social and cultural perspectives. In order to do this, probing questions must be answered in a qualitative interview leading to multi-layered responses that include information concerning social factors, knowledge, coping strategies and symptom narratives. Information relating to a patient's view leads to a better understanding of their illness, its cause and meaning, treatment regimes and the recovery process. Individual variations in a patient's conceptualisation of the illness have been identified as important in determining help-seeking and treatment behaviours, preference of treatment, ability to cope and quality of life,[12,13] and these may differ substantially from conventional medical concepts held by health professionals but must be taken into account in the clinical process. This is particularly important in developing countries where pluralistic health systems are commonplace and pose challenges to health planning because the socio-cultural context of the illness leads to 'traditional' therapy rather than focusing on conventional medical concepts.

### Explanatory Models, Mental Distress and HIV infection

HIV infection is associated with psychological problems and psychiatric disorders, and in a study carried out in Zambia the effects of HIV infection on mental distress were demonstrated to be direct (biological) and indirect (psychological)[3]. Psychological effects were prominent and socially patterned, and females with poor socio-economic position and poor social support were at highest risk of developing mental distress. Furthermore, evidence suggests a heightened risk of contracting HIV infection among individuals suffering from mental disorders[1,15]. Mental distress is relevant to HIV disease progression as it can lead to decreases in CD4 T lymphocytes and increases in viral load, and is associated with an increased risk of clinical decline and mortality[16]. Mental disorders can mediate delayed help-seeking and therefore diagnosis, and poor compliance in terms of taking prescribed medication, and can predict individuals dropping out of HIV-risk reduction programmes. Although there are many data concerning the association between HIV infection and mental distress and the importance of explanatory models of mental distress, there has been little research concerning explanatory models for mental distress during HIV infection. There is a high prevalence of HIV and mental distress in the general population of Zambia [3] but only one study has investigated explanatory models of mental health among low-income women and health care practitioners[9]. This study revealed that the most commonly-used phrase among women to define and explain mental health problems was "problems of the mind" but only physical symptoms were defined as an illness. The results indentified socio-economic standing and the home environment as key factors in mental distress, particularly the quality of marital relationships. The study concluded that greater awareness of explanatory models was essential and would have beneficial effects on the formulation of health policies concerned with mental health[9].

The present study considers the importance of explanatory models for improving the provision of mental health services. As far as we are aware, there have been no studies in Zambia or elsewhere in the sub-Saharan region to investigate explanatory models for mental distress during HIV infection. Therefore, this study compared explanatory models used by HIV-infected individuals, uninfected individuals and local health care providers (traditional healers and health professionals). We aimed to explore the narratives of the personal lives and health of individuals using the following key research questions: 1. what are the explanations used for mental distress in Zambia? 2. Are the explanatory models used by people attending primary health care clinics consistent with those of health professionals working in these institutions? 3. What is the significance of explanatory models in mental health in Zambia? In addition, we aimed to build a theoretical model linking the perception of illness to the course of the illness and resultant coping mechanisms, based on a locally validated self-reporting questionnaire. (SRQ-10)

## Methods

### Setting and study design

This research was part of a larger study aimed at validating the use of SRQ-10 as a screening instrument and to assess mental health changes in people living with HIV/AIDS (PLWHA)[3,4]. We assessed primary health seekers at four primary health care centers in Lusaka, Zambia between December 2008 and May 2009. The clinics were purposely selected within the city of Lusaka; two were situated in very high population density areas (Kalingalinga and Mtendere) and the others were situated in a medium density area (Chilenje and Chelston). The residents of these areas speak a number of languages but predominantly English and Nyanja.

### Sampling procedure

In the validation study, a sample of 400 clinic attendees aged 16 years and over was asked to participate in the study between January and March 2009. Each clinic was sampled randomly on selected days, three times each week. On the selected day, interviews were conducted with consecutive clinic attendees in the outpatients department. The purpose of the study was explained to each participant by research assistants and verbal consent was obtained. Details of the methodology are published elsewhere[3]. To fulfill the aims of the explanatory models for the mental distress study, a sample of 28 informants was taken from the validation study. This sample consisted of 14 informants who were HIV negative and 14 who were HIV positive. Of the 14 informants who were HIV-infected, eight were not eligible for ARVs and the remainder had been taking ARVs for varying durations. An effort was made to balance the gender and age distribution across these categories. Household in-depth interviews were conducted to gather additional and detailed data concerning the explanatory model for mental distress in Zambia. Eight eligible health professionals working in a mental institution were interviewed at their respective public health facilities. Three identified indigenous healers who reported treating mentally distressed people were interviewed.

### Data collection

Data were collected using a semi-structured questionnaire and serial in-depth interviews. The questionnaire contained a section comprising questions pertaining to socio-demographic factors such as sex, age, marital status and number of children, education, employment, religion and questions assessing socio-economic position. The other section contained questions concerning mental health. This information was extracted from the database of the main Validity study[4-6]. Eligibility for participation was based on the participant's HIV status and being mentally distressed, as determined using a locally validated Self-Reporting-Questionnaire-10 (SRQ-10), a 10 item questionnaire containing two domains, namely depressive symptoms and somatisation. The SRQ-10 is based on a dichotomous response answer system (Yes/No) to the questions presented in table 1. Each symptom was weighted according to severity based on the DSM-IV criteria, with more severe symptoms ranked higher than less severe ones[4-6]. The raw weights were summed up in a transformed summative index ranging from 0-20. A cut-off point of >7/20 (for mental distress cases) was selected on the basis of the DSM-IV requirement of five or more symptoms under the headings: thoughts of suicide, loss of interest or pleasure, and depressed mood, which would represent a change in a participant's previous functioning[4-6].

**Table 1:** SRQ-10 diagnostic symptoms

A. Thoughts of Death	*Has the thought of ending your life been on your mind?*	
B. Loss of interest or pleasure	*Is your daily life suffering?*	
	*Are you unable to play a useful part in your life?*	
	*Do you find it difficult to enjoy your daily activities?*	
C. Depressed mood	*Do you sleep badly?*	
	*Do you cry more than usual?*	
	*Do you have difficulties deciding?*	
	*Are you tired all the time?*	
	*Do you often have Headaches?*	
	*Is your digestion poor?*	

Chipimo PJ, Fylkesnes K.***Mental distress in the general population in Zambia: impact of HIV and social factors*. **BMC Public Health. 2009; 9 (298). doi:10.1186/1471-2458-9-298

Informants classed as mental distress cases underwent further qualitative interviews to elicit the explanatory models. A modified, adapted and contextualised interview schedule developed by Kleinman [17] was used to elicit the explanatory models for mental distress (Table 2). To compare the explanatory models, this interview schedule was administered to all groups in the study (HIV negative individuals, HIV positive individuals and health care practitioners). The interviews with health care practitioners were conducted in English and designed to draw upon their experience of attending to patients and eliciting explanations for the causes of mental distress in the study population profile.

**Table 2:** Kleinman Interview schedule for explanatory models

1.	*What do you call your problem? What name does it have?*
2.	*What do you think caused the problem?*
3.	*Why do you think it started when it did?*
4.	*What does your sickness do to you? How does it work?*
5.	*How severe is it? Will it have a short or long course?*
6.	*What do you fear most about your illness?*
7.	*What are the chief problems your sickness has caused for you?*
8.	*What kind of treatment do you think you should receive? What are the most important results you hope to receive from the treatment*

Kleinman A. ***Patients and healers in the context of culture***. Berkeley, CA: University of California Press. 1980.

### Data analysis

Socio-demographic characteristics were extracted from the database of the main study[4-6]. Tape-recorded in-depth interviews (IDIs) were transcribed verbatim either directly in the case of interviews conducted in English or from the language of the interview (Nyanja or Bemba) to English. Initial analyses were carried out manually using the code sheet, which is an interpretative approach to identifying common themes in a data reduction strategy[18]. A sheet containing all phrases representative of the five models was created and the names of models were used as codes. These standardized codes were assigned to the same or similar phrases. In this case, phrases refer to responses given according to the question posed. Common phrases were grouped together and placed under the same or similar sub-theme. Themes described the codes, and the codes were representative of names of models. Themes that were grouped together provided unique and contrasting features of the narration of the symptoms[18]. To confirm consensus relating to codes assigned to phrases, two authors independently produced code sheets with identical standard codes, and switched them. This allowed agreement and disagreement about standard codes assigned to phrases to be identified. Where there were disagreements, the phrase was re-coded. Therefore, atypical data were selectively discarded, allowing the study to focus on the most common answers. To ensure validity of responses, transcripts were read and re-read. The frequency and occurrence of common phrases was noted. To ensure reliability of phrases, occurrences and frequencies of common phrases within single IDIs and across IDIs were compiled and noted.

The data were entered in SPSS to enable basic statistics such as frequencies and cross tabulations to be analyzed electronically. A theoretical taxonomic model was developed to aid the classification of contrasting symptom narratives. The narratives were grouped into representational categories that mirrored the contrasting models between the role of social circumstances, worries and emotional experience. A link was drawn between the perceptions of the symptoms and attitudes towards treatment and coping strategies.

## Results

Twenty-eight informants (13 females, 15 males), who met the symptom criteria for Mental distress (Table 1) and consented, were interviewed. An effort was made to balance the male to female ratio despite clinical demographics demonstrating that more women than men seek health care. The combined mean age was 32 years (35 years male, 29 years female) and the age range was 19-56 years. Of the 28 participants, 50% were HIV positive (eight males and six females) and 50% HIV negative (seven males and seven females). HIV positive informants were divided into two groups depending on whether they were receiving ARVs. Nine informants were HIV positive but not receiving ARVs (four male, five female) and six were receiving ARVs (three male, three female).

Table 3 presents the factors associated with mental distress and the most commonly associated symptoms; this is a summary of the broad categories of explanations given by the informants for their mental distress symptoms. The numbers in each category are higher than the sample size as several informants related more than one symptom in describing their experiences.

**Table 3:** Factors associated with Mental Distress as identified by the informants

**Category of factor**	**Frequency**	**Symptoms/comments**
Worries about money	51	Concerns about rent, day-to-day living, school fees
Problems of the mind	47	Recurrent headaches, sleeplessness, unhappiness, trouble thinking, loss of appetite, night mares
Unknown cause of symptoms	32	Most common among informants not acknowledging symptoms as an illness or as mental distress, suggested witchcraft
Relationship with spouse and family members	26	Commonest among women, included crying more than usual, unhappiness, headaches, sleeplessness.
Ill health	24	Sleeplessness, daily life suffering, inability to play useful role in life, tiredness
Low self-esteem	16	Worthlessness, loss of interest, unhappiness, crying more than usual, difficulty enjoying daily activities, experience of stigma
Recent life events	6	Bereavement, divorces, newly diagnosed with chronic disease including HIV. Included symptoms of restlessness, sleeplessness, trouble thinking, headache, unhappiness

### The Explanatory Models

To aid understanding of the contrasting models for mental distress in the study, taxonomic categories were developed and respondents were classified into one of these representational groups: social, biological, psycho-social and situational models. Attributes of these models were closely interrelated, but certain features and aspects belonged to specific groups as demonstrated in table 3.

#### 1. Social Model

Informants in this model said that their symptoms were due to social events in their lives, either single episodes or long-term stressors. In some cases, informants described multiple sources of social stressors. The social narratives were closely related to recent life events that had traumatized the informants, causing repeated somatic symptoms.

##### Social Narratives

A 48 year old unemployed man with six children ascribed his symptoms to the lack of a job.

*"I only went up to grade seven in school because my parents died early and so there was no one to educate me. My life has been tough. I am 48 years old and been married twice. I have six children spread in two homes. The biggest problem I am facing is I do not have a job and I cannot educate my children. My life is so unstable and all I do is piece work. How can I pay for two homes? How can I pay for the house and buy food? When I think like this I get headaches and I cannot sleep. I have no taste for food in my mouth. I am suffering a lot*.

Another 29-year-old married woman with four children said her problems had started when her husband died.

*"I have a lot of thoughts, often I sit and cry by myself and I cannot remember the last time I had a good night's sleep. I feel so alone. I am the one who has to think about where the money for food and rent is going to come from. When my husband was alive, this was automatic. I pray that his soul has found a better place. I sell some tomatoes by the roadside but this does not make any money at all. I am already one month in arrears for rent and I do not get any help from my in-laws. I worry a lot about how I am going to take care of my four children and about how I am going to pay for their school fees. My mind comes to a stand still and I feel like such a worthless person"*.

The results showed that mental distress was somewhat insensitive to gender. However, certain social stressful situations had more effect on women than on men.

#### 2. Biological Model

This model was closely related to the social model. The narratives in this category ascribed the symptoms to physical ailments currently being experienced. Unlike the social models, the stressor (physical illness) was a single entity. Expressed worries or symptoms were a direct result of the physical illness. Therefore, the body was seen as mediating the social stressors, which were expressed as symptoms of mental distress. This model predominantly comprised informants who were HIV positive and included individuals with other long-term medical conditions such as hypertension and diabetes.

##### Biological Narratives

A 29-year-old unemployed man with 10 years of education said his symptoms were due to HIV and worsened by worries concerning his family (married with two children).

"As I told you, I am HIV positive and I have been on ARVs for three years now. Yes, I often have stomach problems and trouble sleeping. Sometimes my hands shake and I wake up abruptly at night due to bad dreams, but I do not know why. I think that it may be due to the HIV since I have been HIV positive for seven years now. I think these are complications of the illness, maybe the virus is in my brain now. I am not worried about my health, but I do get worried often about the future of my children who will suffer and live on the streets when I die. I have repeated headaches when I think too much about this and I lose sleep. I am currently unemployed and occasionally get paid for fixing people's radios, but I am always short of money for food and paying rent. If this is happening now, imagine after I am buried six feet under."

A 55-year-old self-employed man with 14 years of education ascribed his symptoms to hypertension (BP). He is married with five children.

*"BP is a bad disease as you know. They call it a silent killer. I could be talking to you right now but maybe I'll collapse the moment I stand up... Headaches are my daily problems, I think it is these BP medicines. Also they say BP itself cause headaches, I also sleep very badly because I sweat a lot at night and have bad dreams. These are worse when I have a lot on my mind. I worry everyday that I will have a stroke... I am not worried about dying because I know my family will live a good life, but if I have a stroke it means that my wife and children have to change their lives to care for me. Maybe I will not be able to walk or feed myself, maybe my wife will have to wash me and take me to the toilet. I don't want to be a burden. These thoughts stress me a lot and I even get depressed"*.

The results revealed that worry concerning the lives of children and spouses if the individual died was a direct contributor to headaches and sleeping badly.

#### 3. Psychosocial Model

Attributes in this model emphasize the role of psychological stressors in mental distress. Although the stressors were a single entity, they manifested themselves as a psychosocial dimension. However, these narratives focused on themes of self-blame, personal failure and poor self-esteem.

##### Psychosocial narrative

A 26-year-old married woman with 17 years of education attributed her symptoms to her failure to have children.

"I am well educated and have a good job, but unfortunately my marriage is on the rocks and I think it is entirely my fault. You see the Good Lord has given me everything else but the ability to have children. When I dwell on this I often cannot think straight, I am not even sure how to describe my state of mind. I get so anxious and depressed. I feel I am a worthless person and my time here on earth is meaningless. I always feel like something bad is going to happen. I often avoid going to kitchen parties or women's meetings at work. They all have kids and I know they talk about me. My husband has two children with another woman and he spends most of his time there and comes home only to change clothes. Once I confronted one of the other women and she shouted at me and said I was not a woman. Those words have never left my mind."

A 34-year-old woman working as a secretary attributed her symptoms to her physical disability. The physical disability was mentioned as the cause of the distress, although the psychosocial reality was emphasized in the narrative, making it more salient than the physical disability.

"... I had polio when I was young and it left me in a bad way... I am 34 years now and slowly getting old. I have never been married and don't have any children. Last year I had a boyfriend and I hoped that the year would not end before I got married but this didn't happen. Now I am just an object of pity. Both my young sisters are now married and have children. I have nothing to show at family gatherings. I always just go alone. I think this depresses me and I wonder what this entirely means."

Results demonstrated that suffering from certain conditions that left permanent scars on the body hindered access to social agreements such as marriage and hence contributed to mental distress. An inability to bear children was a factor contributing to mental distress in women.

#### 4. Situational Model

In this category, symptoms were the result of a stressor that would result in a change in the respondent's life. Informants in this category viewed their stressor as representing 'the end of the road'. For example, this category included a 22-year-old university student who had discovered that he was HIV positive less than a week before the interview.

"This is a tragedy to me, I am young and my life is ruined before it has started. I stopped going to class because there is no point. I don't even know how to tell my parents. I have not slept in a week. I tried to drink beer so that maybe I sleep, but the beer tastes sour. This is pressure. I have no appetite, my mouth is sour. I sweat at night and I have a pounding headache."

The results demonstrated that HIV positive results contribute to mental distress.

### Perspectives of health professionals and healers

Many responses given by health providers were identified under the theoretical explanatory models, confirming consensus-driven attributable causes, treatment-seeking and response behavioural challenges from institutional and community perspectives. Health professionals reported that HIV infection was responsible for approximately 40% of institutionalized mentally distressed patients. When providers and healers constructed mental distress using reports from users, witchcraft and stigma emanating from HIV positive results emerged as major contributors to symptoms of mental distress.

*"Because when you are told that you are HIV positive you think that you will die any time, so they have that fear." *(Indigenous healer, male 39 years old, six years experience of treating HIV-infected persons.)

*Most of the time these people are in denial. They say that I am not HIV positive, me I am not mentally sick, I have been bewitched, so it's very difficult. Some of them deny they are restless, they are... they cant see... you can see that this person is not as he used to be, moving up and down, shouting, me I am not sick, even if you are not talking about the... the topic they are just guilty somehow they have feelings like maybe these people they know that I am HIV positive. (*Female health professional, 43 years old, 11 years of service.)

*Yes. Some they come here and say "Bana lilowa" (they have bewitched me) "Nima shabe" (It is evil spirits)*. *(*Female health professional, 42 years old, 18 years of service)

*Okay, I have forgotten one thing, some of them they say he slept with someone who aborted, she has "kapopo" you know what I mean*. (Female health professional, 42 years old, 18 years of service)

The results revealed that despite noticeable changes in mood including social withdrawal and mood swings in a family member, health services were only sought when physical violence or suicidal tendencies developed. Until that point, most were home-bound.

*Most of the time, it's when they become violent. They are short tempered or start threatening that they would kill themselves, breaking things, things like that. That's when they rush there*. (Male health professional, 42 years old, 18 years of service)

Gender dimensions were noted when attributable causes to mental distress were assessed. Females were more likely to report social problems including stigma owing to HIV status, whereas males attributed causes to ARV treatment. In terms of treatment at home, all providers reported that predominantly women took that responsibility.

*A close relative, someone they trust, someone who has been caring for them in the hospital. It's usually women*. (Male health professional, 42 years old, 18 years of service)

When age was isolated, there was agreement between professional providers and healers regarding the age group (15-35 years) of people frequently presenting with mental distress symptoms due to HIV. Furthermore, positive responses to treatment were obtained from professional providers and healers.

*We traditional healers, we use leaves and roots, so we have the roots that we give them and sometimes we send them to test and most of them are responding well to treatment *(Indigenous healer, male 39 years old, six years practice with HIV positive persons.)

Despite the reported treatment response, integration of these persons remains a challenge.

*That one is a problem and is still difficult. A long time ago there used to be community psychiatry outreach where we would follow up patients to see how they were integrated but that was causing more problems. Even from their own families, they would say look he has come to visit that one who wafuntila (that one who was mentally distressed), he has not recovered fully. So that was causing too much stigma on those who may have completely recovered. That was the other reason causing relapse. Family members would keep reminding them that "Ichi ni cho funta" (this one is mentally distressed). It's a big problem*. (Male health professional, 42 years old, 18 years of service.)

### Significance of explanatory models: Health-seeking and coping strategies

A large body of research has documented a strong relationship between stressful situations and mental distress. Therefore, the relationship between coping and mental distress holds specific interest in both HIV-infected and uninfected individuals. Coping mechanisms can be described as the sum total of ways in which we deal with minor stress, major stress and trauma. Many of these processes are unconscious, others are learned behaviours, and some are skills we consciously master to reduce stress and intense emotions such as depression. However, not all coping mechanisms are equally beneficial, and some can be detrimental.

### Health seeking and coping strategies among uninfected individuals

Informants in the *Social Model *were able to identify the cause of their symptoms and relate the cause directly to one or multiple social stressors. They relayed that they were unsure how long the stressors would last, that they did not recognise their symptoms as a disease entity and saw no need to seek medical attention. They stated that their symptoms would disappear once the stressors were removed and/or if they found a job to relieve the financial strain. Their coping strategy was based on the hope that things would improve.

"... I do not think my symptoms are an illness, its just problems with life. What can a doctor do for me unless he gives me a job to work at his house? I am just unfortunate but I am sure, God willing, I will find a job soon so that I can pay for my rent, buy food and take my children to school."

In the *biological model*, informants viewed their symptoms as part of the whole disease process and they were eager to consult medical personnel for treatment. However, they did not relate their symptoms to mental distress but "worries" for which they did not need psychotherapy or psychotropic medication. They said that their symptoms would not disappear permanently but have a recurring pattern. Their coping strategy was one of longing for longer symptom-free periods.

"As you know "sugar disease" (Diabetes) has no cure. It is here to stay. The doctors and nurses have told me what I should eat and what I shouldn't. The problem is, sometimes, even if I follow their advice, I still find myself admitted to hospital. Since I am self employed, it means for the period I am sick, there is no money coming into the house to pay my "nkongole" (debts). My family upkeep then becomes tricky... There is little I can do about this disease but to follow the doctor's advice and pray to God that I don't fall sick often, so I can care for my family."

Informants in the *psychosocial model *did not recognize their symptoms as an illness. They described their symptoms as normal reactions to events in their lives, but were willing to consult medical professionals. They admitted that psychotherapy would help but were very skeptical about the role of psychotropic medication in alleviating their symptoms. Hopelessness was identified as an important factor in this model. They remarked that their symptoms would run a chronic course with no hope of alleviation. The coping mechanism identified was religious faith and/or the hope of meeting somebody who would accept them for who they are.

"God never gives everything that a person needs. He always leaves out something. If he gives intelligence, he takes away beauty; if he gives you beauty, then he takes away the riches. There is nothing I can do about my situation. I guess I need to undergo some counseling because I think I am depressed... I cannot commit suicide because then I would be ungrateful for the life God has allowed me to have. I just hope that God has one more gift for me and I pray hard for that."

### Health seeking and coping strategies among HIV-infected individuals

HIV positive informants fitted into the biological and situational models. Their symptoms of mental distress were ascribed specifically to their HIV status. However, some differences were noted in the health seeking and coping strategies. The differences were a function of how long they had known that they were HIV positive and if they were receiving ARVs. Informants who were newly diagnosed and those who had known of their HIV+ status longer but were not receiving ARVs had a high degree of hopelessness.

"My life is not worth living, I do not know how this happened. I feel worthless; I cannot contribute to my children's future... I will die soon. I don't even think these new medicines work. People say the medicines themselves can kill because of all the side effects... why take them if they are just prolonging misery... The whole disease is just stressing, it has left me helpless. When I see the disease finally cripples me and my hair falls off, everyone will know that I have AIDS and will say I have been sleeping around... it's just better to avoid everyone and stay alone."

In contrast, informants receiving ARVs had a lower degree of hopelessness. They recognized their symptoms as an illness requiring medical attention in the form of psychotherapy. However, they did not see the immediate benefit of psychotropic medication, and informants who had been very sick and/or had opportunistic infections prior to starting ARV expressed optimism about their lives and future. Most of the informants in this category did not view psychiatric consultation as necessary or appropriate for their situation, though they admitted that psychotherapy would be of help.

"You should have seen me three months ago. I was finished, I had had TB three times and I was half my weight. I thought I was going to die. I had lost all hope and every time I saw my kids I felt so low and hopeless. But now I am much happier and I have hope that I will see my children grow to adulthood. All this in just six months of taking ARVs... Yes I still get headaches when I think a lot, especially that life is rough and money is a big problem. I get a lot of worries about it but these come and go... At least I am much better now and when I feel bad I just go and see the clinic people... the counseling works and we get a lot in the support group meetings. After these meetings I often go home in a better mood."

### Comparison of explanatory models: Perspectives of the patient, health-care professionals and healers

Table 4 presents a summary of the comparative models used by the patients and health care providers. A general measure of agreement exists between explanatory models of the study groups. The experience of mental distress among patients appears to have been governed by problems relating to socio-economic problems (poverty), particularly problems in the home (marital problems). However, occasional differences were noticed. Male respondents emphasized economic problems more, while female respondents emphasized social problems (marital, violence in the family, alcohol abuse by spouse). Female respondents mentioned economic problems as a secondary effect of separation, divorce or being widowed. Additional explanatory models were noted in the presence of chronic illness including HIV, hypertension and diabetes. In these circumstances, explanatory models emphasized the role of the physical illness in the experience of mental distress. Perception of the cause of the symptoms, expectations of the course of the illness, severity of the symptoms, family support and presence of stigma, were all predictors of health-seeking behaviour.

**Table 4:** Summary of comparative explanatory models used by patients and health care providers

Components of explanatory model	HIV -	HIV +	Health care providers	Comments
1. Name given to symptoms	-Problems of the mind	-Problems of the mind -Depression and stress	-Depression, stress -problems of the mind	-Occasional differences exists in the name given to symptoms between the health professionals and the traditional healers
2. Cause of symptoms	-Poverty, marital problems, -witchcraft	-Worries about course of disease, worry about future of family - HIV infection	-Social-economic problems, intercurrent illnesses - bad spirits	Witchcraft was cited as a cause among some the traditional healers. HIV infection was cited as a cause mostly among HIV+ not on ARV's
3.Common symptoms of experience	Headache, sleeplessness, poor appetite, worthlessness, crying	Worthlessness, loss of hope. Somatic symptoms	- Somatic symptoms, - Social withdraw	Significant differences noted based on HIV status and (if on) duration on ARVs
4. Greatest fear about their experiences	- Worries about future of children, - death, - disability from illness, - not ever getting married	- Children's future, - Death, - Stigma from relatives and friends - Course of illness	- Future of family, - Death, - Stigma from family and friends, - Short life expectancy	- Worries about future of family were the majority. Short life expectancy was next and the newly diagnosed worried more about stigma
5. Severity of experience	- Severe but can have a remedy	Extremely severe, no way back, hope for the best	Severity depends on other circumstances such as poverty and family support	Severity depended a lot on level of adjustment to HIV status and severity of circumstances
6. Choice of treatment	Clinic, pain killers, majority no treatment	Clinic, counselling, - Religion, - no treatment	- Counselling, - Exorcism, - Support groups, family involvement, - Antidepressants	Choice of treatment depended on what was thought to cause the problem
7. Factors leading to choice of treatment	Severe symptoms (headache, tiredness, loss of sleep)	Severity of symptoms, - family support, - Disclosure	Severity of symptoms, cause of symptoms, previous failed consultations, Family involvement	Factors associated with treatment options were, perceived cause of illness, family support.
8. Course of symptoms and alleviating factors	- Short course, - Finding cure, - Solution possible (finding a job, improved marital relationships), may have a chronic course	Chronic course, - ARVs, - Family support, social support groups, - Prayer - No hope	- Can be modified by counselling and medication - Support groups - Traditional medicine -	- Course of illness associated with perceived course of illness, ARVs - Alleviation of symptoms dependent on adjustment to illness, medication and counselling

A broad consensus was apparent among the health care providers, although there were some clear differences. Health care providers agreed that symptoms were problems of the mind mediated by socio-economic problems. They were in agreement that these symptoms required some form of intervention (medication, prayer or exorcism) in order to alleviate the suffering of the patient. They agreed that HIV poses special circumstances and that it causes much distress. However, there were occasional differences in emphasis on the cause, course of illness and preferred treatment (Table 4).

However, a greater difference was evident between the patients and the health care providers. Health care providers had a predetermined cause and effect pathway, either: (1) social circumstances leading to mental distress (stress, depression) and in turn mental distress symptoms; or (2) bad spirits (witchcraft) leading to mental distress symptoms. Patients had a somewhat different narration of the illness experience in the different models. HIV positive individuals (regardless of ARV history) and those with chronic medical conditions established a cause and effect pathway, but HIV negative individuals did not; they had a more narrative and experience-based understanding of their illness. They did not readily attribute their mental distress symptoms to an illness entity but to life's problems and a few of them entertained witchcraft as a cause. This impacted profoundly on what treatment choices they made.

## Discussion

This paper outlines the findings of a qualitative study nested within a cross-sectional validation study investigating the importance of explanatory models for improving the provision of mental health services, particularly for HIV-infected individuals. Explanatory models used by HIV-infected and uninfected individuals were elicited and compared to those of local health care providers. The relationships between these explanatory models, health seeking behaviour and coping strategies were investigated. The salient findings of this research are that patients without chronic illness, who are identified as having mental distress, express these symptoms through an array of somatic symptoms that they attribute to social disadvantage and strained family relationships. In contrast, those with chronic illnesses such as HIV attribute their experience of mental distress to the disease process. However, their symptoms are perpetuated by existing social circumstances and worry concerning the future. HIV infection added strain and this contributed to the onset of symptoms and compounded other existing social stressors. Health-seeking and coping strategies were determined by the cause of the illness, its perceived course, family support and perceived duration of illness. The explanatory models of the patients and the health care providers did not differ as much as expected. While health care providers had a predetermined structure in which they recognised mental distress, patients had an illness experience-based rationalisation of symptoms. The findings suggested that health care providers were unfamiliar with how the patient's explanatory models affected health-seeking and coping behaviours, similar to the findings of previous studies.

The constellation of somatic presentations in this study has previously been reported in Zambia [3,4,9] and other developing countries[19-21]. However, the diagnostic significance of these symptoms for identification of mental distress is not universal. They may vary widely among societies according to the burden of disease, gender perspectives and the societal perception of the symptoms. For example, some societies would emphasize fatigue [22] while others would emphasize headache[4]. Somatic symptoms appear to be more consistent universal indicators for mental distress across cultures, and this has been demonstrated in this study where somatic complaints were a central feature of the narratives.

Causes of mental distress symptoms were somewhat different in the taxonomic models illustrated. However, two main causes stood out; with the exception of the biological model, economic difficulties and difficulties in marital relationships were most cited. The informants conceptualised their distress as a direct consequence of poverty and attached no significance to a biochemical cause. The more adverse the social circumstances, the worse the symptoms; this finding has been confirmed by other studies[10]. In the biological models (HIV and other chronic illness), there was a direct link between the illness and the symptoms. However, the symptoms were compounded by social difficulties and stigma. Several studies examining the relationship between HIV and psychological variables have demonstrated that patients well-adjusted to their HIV-positive status tended to have lower levels of mental distress and expressed hope[23].

Gender is a critical determinant of mental distress and is closely related to course, care and support. Gender determines the differential power and control men and women have over the socioeconomic determinants of their mental health, their social position, treatment choices and their susceptibility and exposure to specific mental health risks[24]. Women appear to be affected to a greater extent than men across different countries and different settings. Women present with an earlier age of onset of symptoms, a higher frequency of somatic symptoms but less severe illness[24]. Men have a protracted course of illness and they exhibit poor social adjustment and a poorer long-term outcome[25]. Gender-specific risk factors for mental distress that disproportionately affect women include co-morbid circumstances such as gender-based violence, low income and income inequality[26]. Other factors include severe life events that cause a sense of loss, inferiority or humiliation. In addition, women have a responsibility to care for others[27,28]. Pressures created by these multiple roles combine and account for the poor mental health of women. A positive correlation has been reported between the frequency and severity of these social factors and the frequency and severity of mental distress in women[25]. Gender differences exist in terms of patterns of seeking help for mental distress, with women more likely to seek help from and disclose mental health problems to their primary health care physician than men[29]. Therefore, gender-specific determinants and mechanisms that promote and protect mental health and foster resilience to stress and adversity should be emphasized.

Research investigating the psychosocial impact of HIV has demonstrated that the level of mental distress is high among HIV-infected individuals,[3] particularly those recently diagnosed or who have developed opportunistic infections. Several intervening reasons for the onset of mental distress have been discussed, ranging from psychosocial to biological factors. Evidence has linked effecting coping styles to better compliance with ARVs and positive health-seeking tendencies[30,31]. Furthermore, effective health-seeking is associated to a better quality of life, reduction in risk-taking behaviour, and higher total lymphocyte and natural killer cell counts[32]. Therefore, it is imperative that the relationships between coping, treatment choices and psychological morbidity are elucidated. The findings presented herein confirm results from previous studies and demonstrate a significant association between the severity of mental distress, maladaptive coping strategies and poor health-seeking behaviour, particularly among the newly diagnosed.

There are a number of limitations to this study including its qualitative nature, which limits the findings to statements concerning association and not causality. However, the main aim of this study was to describe the explanatory models for mental distress among HIV-infected and uninfected individuals, and how these are associated with coping mechanisms and treatment preferences. This study was conducted in the capital city of Zambia, which has a comparatively high concentration of local and international institutions that support improved quality health services. Furthermore, the urban population has higher educational attainment than rural populations and easier access to private and public healthcare systems. Therefore, generalization of these findings should be limited to settings that have similar social organizational and economic structure as it is unknown if the same model would be applicable in rural parts of the country. A validation study or further research carried out in a different setting and culture with a larger sample size would be useful to confirm the findings of the present study.

## Conclusion

The results of this study support the findings of studies carried out in other developing countries that emphasize the role of social context for understanding mental distress. The patient's conceptual perspective of mental distress is rarely studied and this research demonstrates that the patient's models of illness may differ somewhat from those of health care providers. Their explanatory models are consistent and coherent, and appear to be associated with health-care-seeking behaviour and coping strategies. Therefore, provision of medical treatment should take into account the patient's explanatory models to generate a joint treatment plan. The results in this study suggest that a balance between the professionals' and patients' models is particularly important among HIV-infected individuals. Therefore, we recommend the use of contextualised conceptual models as defining clinical features for understanding the conceptualisation of the clinical syndrome of mental distress for clinical and public health interventions.

## List of Abbreviations

SRQ-20: Self-Reporting-Questionnaire 20; SRQ-10: Self-Reporting-Questionnaire 10; SCL: Symptom Check List; DSM-IV: Diagnostic and Statistical Manual of Mental disorders, fourth edition; HIV: Human Immunodeficiency Virus; PLWHA: People living with HIV/AIDS; ARV: Antiretroviral drugs; IDI: In-depth Interviews; PHC: Primary Health Care; EM: Explanatory Models

## Competing interests

The authors declare that they have no competing interests.

## Authors' contributions

PJC, MT and KF contributed to the analysis and drafting of the manuscript. PJC and MT further contributed to the design and conduct of the study. All authors contributed to the critical revision and final approval of the manuscript.

## Pre-publication history

The pre-publication history for this paper can be accessed here:

http://www.biomedcentral.com/1472-6963/11/7/prepub

## References

[B1] Murray CJL Lopez AD press HU The global burden of disease and injuries series: A comprehensive assessment of Mortality and disability, Injuries and Risk factors 1990 and projected to 2020 1996 1 Cambridge, MA, USA

[B2] Mathers CD Loncar D Projections of global mortality and burden of disease from 2002 to 2030 Plos Medicine 2006 3 442 17132052 10.1371/journal.pmed.0030442 PMC1664601

[B3] Chipimo PJ Fylkesnes K Mental distress in the general population in Zambia: impact of HIV and social factors BMC Public Health 2009 9 298 19689799 10.1186/1471-2458-9-298 PMC2744699

[B4] Chipimo PJ Fylkesnes K Comparative Validity of Screening Instruments for Mental Distress in Zambia Clinical Practice & Epidemiology in Mental Health 2009 1 6 4 15 10.2174/1745017901006010004 PMC2858522 20498698

[B5] Araya R Wynn R Lewis G Comparison of two self administered psychiatric questionnaires (GHQ-12 AND SRQ-20) in primary care in Chile Soc Psychiatry Epidemiology 1992 27 168 173 10.1007/BF00789001 1411744

[B6] Strand HS Dalgard OS Tambs K Rognerud M Measuring the mental health status of the Norwegian population: A comparison of the instruments SCL-25, SCL-10 and MHI-5 (SF-36) Nordic Journal of Psychiatry 2003 57 2 113 118 10.1080/08039480310000932 12745773

[B7] Katon W VonKorff M Lin E Bush TJO Adequacy and duration of antidepressant treatment in primary care Med Care 1992 30 67 76 10.1097/00005650-199201000-00007 1729588

[B8] Simon GE Goldberg SD Tiemens BG Ustun TB Outcomes of recognised and unrecognised depression in an international primary care study Gen Hosp Psychiatry 1999 21 97 105 10.1016/S0163-8343(98)00072-3 10228889

[B9] Aidoo M Harpham T The explanatory models of mental health amongst low income women and health care practitioners in Lusaka, Zambia Health Policy and Planning 2001 16 2 206 213 10.1093/heapol/16.2.206 11358923

[B10] Pereira B Andrew G Pednekar S Pai R Pelto P Patel V The explanatory models of depression in low income countries: Listening to women in India Journal of Affective Disorders 2007 102 209 218 10.1016/j.jad.2006.09.025 17074394

[B11] Karasz A Cultural differences in conceptual models of depression Social science &Medicine 2005 60 1625 1635 10.1016/j.socscimed.2004.08.011 15652693

[B12] Kendler KS Explanatory Models for Psychiatric Illness Am J Psychiatry 2008 165 6 695 702 10.1176/appi.ajp.2008.07071061 18483135 PMC2744075

[B13] Eisenbruch M Classification of natural and supernatural causes of mental distress: development of a mental distress explanatory model questionnaire Journal of Nervous and Mental Disease 1990 178 712 719 10.1097/00005053-199011000-00007 2230759

[B14] KLeinman A Patients and healers in the context of culture: An exploration of the borderland between antropology, medicine and psychiatry 1980 Berkeley: University of Califonia press

[B15] Gureje O Mental Health Policy Development in Africa Bullentin of the World Health Organisation 2000 78 4 475 482 PMC2560723 10885166

[B16] Leserman J Role of Depression, Stress, and Trauma in HIV Disease Progression Psychosomatic Medicine 2008 70 539 545 10.1097/PSY.0b013e3181777a5f 18519880

[B17] Kleinman A Patients and healers in the context of culture 1980 Berkeley, CA: University of California Press

[B18] Moser CA Kalton G Social Methods in Social Investigations 1971 London: Heinemann Educational

[B19] Patel V Gwanzura F Simunyu E The explanatory models and phenomenology of common mental disorders in Harare, Zimbabwe Psychological Medicine 1995 25 1191 1199 10.1017/S003329170003316X 8637949

[B20] Patel V Pereira J Mann A Somatic and psychological models of common mental disorders in India Psychological Medicine 1998 28 135 143 10.1017/S0033291797005941 9483689

[B21] Raguram R Weiss MG Cannabasavanna SM Stigma, depression and somatisation in South India American Journal of Psychiatry 1996 153 1043 1049 8678173 10.1176/ajp.153.8.1043

[B22] Pereira B Andrew G Soluchana P Reshma P Pelto P Patel V The explanatory models of depression in low income countries: Listening to women in India Journal of Affective Disorders 2007 102 209 218 10.1016/j.jad.2006.09.025 17074394

[B23] Grassi L Righi R Sighinolfi L Makoui SFG Coping Styles and Psychosocial-Related Variables in HIV-Infected Patients Psychosomatics 1998 39 4 350 359 9691704 10.1016/S0033-3182(98)71323-4

[B24] WHO Gender disparities in Mental Health 2004 Geveva: World health Organisation

[B25] Lindmer LA Bailey A Hawthrone W Folsom DP Gilmer TP Garcia P Hough RL Jeste DV Gender Differences in Characteristics and Service Use of Public Mental Health Patients with Schizophrenia Psychiatric Services 2003 54 10 1404 1409 10.1176/appi.ps.54.10.1404 14557530

[B26] Belle D Poverty and women's mental health American Psychologist 1990 45 385 389 10.1037/0003-066X.45.3.385

[B27] Kaona FAD Tuba M Siziya S Sikaona L An assessment of factors contributing to treatment adherence and knowledge of TB transmission among patients on TB treatment BMC Public health 2004 4 68 1 8 15625004 10.1186/1471-2458-4-68 PMC545081

[B28] Kaona FAD Tuba M A qualitative study to identify community structures for management of severe malaria: a basis for introducing rectal artesunate in the under five years children in Nakonde District of Zambia BMC Public health 2005 5 28 1 10 15792501 10.1186/1471-2458-5-28 PMC1079879

[B29] McAlpine D Mechanic D Utilization of speciality mental health care among persons with severe mental illness: the roles of demographic, need, insuarance and risk Health services research 2000 35 277 292 10778815 PMC1089101

[B30] Namir S Wolcott DL Fawzy FI Coping with AIDS: psychological and health implications J Appl Soc Psychol 1987 17 309 328 10.1111/j.1559-1816.1987.tb00316.x

[B31] Wolf TM Balson PM Morse EV Relationship of coping style to affective state and percieved social support in asymptomatic and symptomatic HIV infected persons: implications for clinical management J Clin Psychiatry 1991 52 171 173 2016252

[B32] Solano L Costa M Salvati S Psychosocial factors and clinical evolution in HIV infection: a longitudinal study J Psychosom Res 1993 37 39 51 10.1016/0022-3999(93)90122-V 8421259

